# 54th Annual Meeting of the Society for Research into Hydrocephalus and Spina Bifida

**DOI:** 10.1186/1743-8454-7-S1-S1

**Published:** 2010-12-15

**Authors:** Hugh Richards, Deborah Sival

**Affiliations:** 1 Neurosurgery Unit, Box 167, Addenbrooke’s Hospital, Cambridge CB2 0QQ, UK; 2 Department of Neurology, University Medical Center Groningen, University of Groningen, Hanzeplein 1, PO Box 30001, 9700RB Groningen, The Netherlands

## 

The 54th Annual Meeting of the Society for Research into Hydrocephalus and Spina Bifida was held at the University of British Columbia, Vancouver, Canada at the invitation of the local organizing committee; Bill Arnold, Doug Cochrane, Paul Thiessen, Judi Haddy, Nicola Valentine, Bev Irwin and Carol King.

Proceedings began on Wednesday afternoon with the Annual General Meeting of Members. This was followed by a reception for all delegates and guests at th e Museum of Anthropology where we were welcomed to Vancouver by Dr. Ralph Rothstein, Acting Head of the Department of Pediatrics, Faculty of Medicine at the University of British Columbia.

The scientific programme was opened on Thursday Morning by a welcome from Dr. Ross MacGillivray, Vice-Dean of Medicine, University of British Columbia, and included sessions on *‘Hydrocephalus’* (Two Sessions), ‘*Urological Problems and Spina Bifida, , ‘Myelomenigocele: Management and Genetics’, ‘Spina Bifida: Sociological Issues’, ‘Choroid Plexus and Molecular Biology’, ‘Shunts and Infections’.* This year we were pleased to have three excellent Invited Lectures, from Dr. Benjamin Warf (Boston Childrens Hospital, MA, USA), entitled *‘Endoscopic Management of Children with Hydrocephalus and Spina Bifida in East Africa’,* from Professor Rima Rozen (McGill University, Montreal, Canada) on *‘Biochemistry and Genetics of folate Metabolism’* and from Dr. Margot Van Allen (Department of Medical Genetics, University of British Columbia, Vancouver, Canada) entitled ‘*Prenatal diagnosis and Folic Acid in the Detection and Prevention of Neural Tube Defects’.*

The Thursday afternoon social programme consisted of a First Nations Salmon Barbeque Luncheon, a coach trip through Vancouver and a Dinner Cruise departing from Coal Harbour and sailing in Indian Arm of Burrard Inlet. The return at dusk gave the amateur photographers some spectacular views of the Vancouver Skyline in the golden light. The final event of the social programme was a gala dinner in Cecil Green House on the campus of the University of British Columbia.

